# Technology supported learning and pedagogy in times of crisis: the case of COVID-19 pandemic

**DOI:** 10.1007/s10639-021-10706-w

**Published:** 2021-08-26

**Authors:** Vian Ahmed, Alex Opoku

**Affiliations:** 1 College of Engineering, American University of Sharjah, Sharjah, UAE; 2 UCL Bartlett School of Sustainable Construction, London, UK

**Keywords:** Engineering education, Covide-19 pandemic, Online teaching & learning, Emergency response

## Abstract

Online teaching within disciplines such as Engineering require experiential learning that equip future graduates with highly intellectual and professional skills to meet the demands of employers and the industry. The outbreak of COVID-19 however, has shifted the academic community into new landscapes that require educators and students to adapt and manage their expectations. Although literature reports on research attempts to study the implications of Covid-19 on the Higher Education curricular, little has been reported on its impact on Engineering Education. This paper therefore uses the theory of Emergency Management Life Cycle (mitigation, preparedness, response, and recover) as a lens to examine the challenges faced by students and academics and coping mechanism during the COVID period. This study adopts a mixed method approach using a case study from the College of Engineering at a Higher Education Institution in the UAE due to the sudden migration to online teaching amid COVID-19. Data is collected through interviews and surveys with both students and instructors on challenges, strategies and online delivery good practices that enhanced students’ learning experience. The results show that, Technology Supported Learning tools are capable of enhancing students’ experiential learning and associated competencies, however there were a number of pedagogical, technological and psychological challenges that faced students and instructors as a result of the sudden migration online, which are likely to play a role in the impediment of the students’ learning cycle, due to the lack of preparedness in response to the state of emergency created by Covid-19. Despite these challenges, the study found that instructors with effective communication skills and teaching style, competent use of technology, flexible, friendly and supportive attitude towards teaching, played a positive role in mitigating for the lack of preparedness in response to sudden migration online. The study also reveals that by overcoming some of the technical challenges such as slow internet connection and interruptions, lessons learnt from the sudden migration to online delivery amid COVID-19, will help create new opportunities for the use of blended learning approaches to meet the needs of the on-going COVID and future online deliveries.

## Introduction

In a dynamic world that is forever undergoing economical, societal, environmental and political changes, the Higher Education sector in general and the Engineering discipline in particular remain under constant pressure to meet the continuous demands of the industry that is in need of highly intellectual graduates with the relevant cognitive and experiential skills (Henard & Roseveare, 2012; McLeod et al., 2017). Such dynamics and demands, paired with the fast growing and rapid advancements of technology at the dawn of the twenty-first century, have been a great catalyst for change in Higher Education. As such, the history and development of Technology Supported Learning tools goes back to in time to the 1950s with a number of studies and initiatives evolving over time to support its development integration in higher education to date (Drage & Evans, 1988; Edelson, 1996; Jantjies et al., 2018; Joshi, 2020; TLTP Projects UK, 2020; Watson et al., 1987). This has resulted in the introduction of new technologies that employ Virtual reality tools and that make use of mobile and wireless technologies, was followed by platforms for Augmented Reality, smart devices, high speed networks and Cloud Computing (Joshi, 2020) (Jantjies et al., 2018).

Therefore, the history and development of Technology to aid the educational process, erases any doubt that technology has the potential to revolutionize the traditional teaching and learning process, enhance the pedagogy of teaching through synchronous and asynchronous modes, eliminate the barriers to education imposed by space and time and dramatically expand access to lifelong learning. Although universities have generally been quick to adopt new platforms of technologies, their utilization of the technology to enhance the teaching and learning process, has been slow for various reasons. Today more than ever, there is a need for a new reform to revolutionize Higher Education practices, while pedagogy is in its most need for the technology to survive in the face of the current ‘Coronavirus’ (COVID-19) global crises, at a time where university students are expected to study at a distance via online modes of delivery. The COVID-19 pandemic has laid bare the challenges of the current higher education system globally especially in the area of digital technology and the need to provide effective training for instructors/academic to prepare them for the rapidly changing education climate (Rashid & Yadav, 2020).There is no doubt that The COVID-19 pandemic forced educational institutions to switch teaching and learning to online and this sudden migration amplified the existing and new challenges of Technology Supported Learning, and there have recently been few publications that draw on some of the lessons learned through the online migration amid COVIDS-19 (Bao, 2020; Ebrahim et al., 2020; Zhang et al., 2020). However, none of these studies have been related to engineering education. Asgari et al. (2021) add that, even though there is existing research on online engineering education, there is little or no empirical research exploring the challenges and factors affecting online engineering education as a result of pandemics.

This paper therefore discusses the role of Pedagogy and Technology in supporting Engineering Education, using the College of Engineering at a selected Higher Education Institution in the United Arab Emirates (UAE) as a case study to evaluate the state of its preparedness due to the sudden migration from its traditional ways of teaching to online teaching via synchronous and asynchronous modes of delivery in a state of emergency amid Covid-19. The paper also examines the challenges it faced instructors and students during the sudden migration online and how the lessons learnt can form a stepping stone toward revolutionizing their educational practices, which can be transferrable to other educational establishment around the word.

## Pedagogy and technology in engineering education

Technology-Supported Learning (TSL) is described as the incorporation of technology into learning environments that can enhance knowledge, skills and attitudes (Wu et al., 2013). Technology Supported Learning is not merely the adoption of software and applications to manage the learning environment effectively, but it is a well-structured tool that addresses the educational aims and objectives of enhancing the student’s acquisition of worthwhile educational objectives by introducing technological devices (Corte, 2001; Zheng et al., 2019). Literature shows that there are broadly two modes of delivery for the Technology Supported learning environment (Synchronous and Asynchronous modes). In the Synchronous mode, a face–to–face environment that entails the simultaneous presence of the instructor and the learner(s) is created. The mode of delivery can take place either via online learning, i.e. use of video conferencing, live chat and instant messaging or in a face–to–face environment, which allow real time interaction for the learners in synchronous online teaching. The environment allows students/instructors to ask questions, share applications, conduct live presentations and surveys, manage group dynamics, share digital whiteboards and also conduct online assessments in real time.

However, the ‘Asynchronous’ mode of delivery allows a convenient environment to the learner, which includes (but not limited to) online material such as; audio and video clips, communication through discussion board and email. With asynchronous mode the learners can work on their own pace and time of the day. Though the instructor input is very different from the synchronous environment such as shorter visits to discussion boards or forums, it allows more valuable and structured feedback to the learners as compared to a single, long session. Thus, a ‘blended’ approach can bring together the advantages of synchronous and asynchronous teaching, into a single experience. On the other hand, a number of studies show that learning through either mode of delivery can only be effective when aligned with the understanding of learning pedagogy and how their use can be utilised to support the different stages of the learning process. The rest of this section will therefore discuss the pedagogy of learning and the important role that it plays in supporting students’ learning within the engineering discipline when coupled with the use of Technology Supported Learning tools.

### The pedagogy of learning

**Fig. 1:**
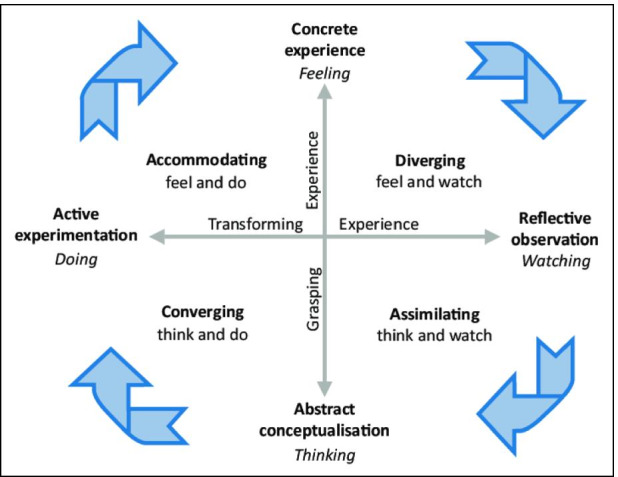
Kolb’s experiential learning cycle. (Source: Kolb et al., 1984)

Pedagogy is defined as an act of teaching which helps teachers’ shapes their actions, judgments, and teaching strategies (Loughran, 2013). The pedagogy of learning takes into consideration learning theory, understandings of students’ learning needs, background and interests of individual students (Loughran, 2006; Shulman, 1987). Literature shows that since the 17th Century systematic, theories of learning have emerged and have been categorized into two broad families; S–R (Stimulus–Response) conditioning theories of the behaviourist’s family and cognitive theories of the Gestalt-Field Family, and that different definitions of learning have risen from such different schools of thoughts and learning theories (Bransford et al., 2006; Leonard, 2002; Schunk, 2012). Although these definitions vary in description, they all agree in principle as explained in this section. Mumford (1980) defined learning from the behaviourists view of learning as a relatively lasting change in performance where “learners know something they did not know earlier and can show how they know it”. Hence, learners are able to do something they did not know how to do before. This theory therefore derives measures of changes in behaviour as a result of the learning process and is a valuable guide to detect the learning outcomes as a result of changes in the learners’ behaviour (learning), with the underlying principles (Pierce & Cheney, 2013; Toker & Avcı, 2015). On the hand, the cognitive theory develops an understanding of how information received from experience is processed and developed into cognitive and intellectual strategies (Bigge & Shermis, 1992), hence, it is through understanding of cognitive theory, that educators can understand how the information gained is processed in the mind of the learners to develop their intellectual abilities. Kolb et al. (1984) however, developed and experiential learning which was based on previous authors such as Habeshaw (1990) and Piaget (1961), emphasizing that the experiential learning process is a cyclic process which can be conceived in four-stages;

According to Kolb the 1st stage of learning is *Concrete experience*, whereby learning can happen either through a completely new experience or a reimagined experience that has already taken place. At this stage, each learner engages in an activity or task, while the key to learning is involvement. Hence, reading or watching in not sufficient to in order to acquire new knowledge, and learners must actively engage in the set task. This stage is followed by *Reflective Observation* and this is when learners should step back to reflect on the task they engaged in. This stage in the learning cycle allows the learner to ask questions and discuss the experience with others and seek feedback. At this stage of the learning process, communication becomes vital, gas it give the opportunity to the learner to identify any discrepancies between their understanding of the gained knowledge and the experience itself. The 3rd stage is *Abstract Conceptualization*, at this stage learner should try to make sense of the gained knowledge in order to draw conclusions from the gained experience by reflecting on their prior knowledge, relating to ideas that they are familiar with, or discussing possible theories with peers. When learners, begin to classify concepts and form conclusions on the events that occurred, they move from reflective observation to abstract conceptualization, which entails interpreting their experience and making comparisons to their current understanding on the concept and modify their assumptions on already existing ideas**.** Finally, *Active Experimentation* is the stage that allows the learner to apply their knowledge in view of the gained experience by applying it into practice and showing its relevance to the exiting situation. Figure 1 shows Kolb’s experiential learning cycle with the four stages integrated in this cycle.

One of the most relevant features of the experiential learning theory is that the direction the learning takes is governed by learners’ needs and goals. Learners seek experiences that are related to their goals, interpret them in light of their goals, and form concepts and test the implications of these concepts that are relevant to these needs and goals. Kolb et al. (1984) argues that, learners can enter the learning process at any stage of the learning cycle, they should really complete the entire cycle for the full learning to take place, and although all of these stages work together, some learners may prefer some components of the learning process over others which is dependent on the learners’ learning style. Hence, some individual may prefer to ‘think and do’ more, while others may prefer to ‘feel and watch’ more. It is for this reason that learners may drop out of the learning process, if the pedagogy of teaching does not support their preference.

Although literature shows that the technology is available and is capable of facilitating the different stages of the experiential learning process (which is the mode of learning for engineering students), this study therefore explores the challenges that faced academics and students due to the sudden migration online amid Covid19 and their likely impact on the learning experience of engineering students based on a selective case study. In recognising such challenges, both educators and students can be better equipped towards a more effective teaching and learning process in times of crisis and beyond. The next section therefore discusses the role of technology in facilitating teaching and learning and highlights the most important challenges cited by literature in view of the use of technology to support Teaching and Learning.

### Technology supported learning for engineering education

Learning from experience is therefore the mode of learning in Engineering, which makes the experiential learning theory relevant to the process of learning in Engineering education and by recognizing dominant learning styles of engineering students, can help recognize the type of competencies that should be enriched for Engineering students and how technology can be used to support these competencies. In support of this, Kolb et al. (1990) studied two different samples of engineering and social workers. The results indicated that professions with a technical or scientific base, such as Engineering, have people with primary Convergent learning styles. Kolb et al. (1990) also adopted previous research results of students’ learning styles, characterized by the subject matter in areas, in a small western college at the University of Illinois and those of American Colleges and Universities. These results show that, in an undergraduate college (of managers who have completed or still in college) the learning style of engineers on average fall in the convergent quadrant, bearing in mind that this may differ between civil engineering, electrical or mechanical engineers.

**Fig. 2:**
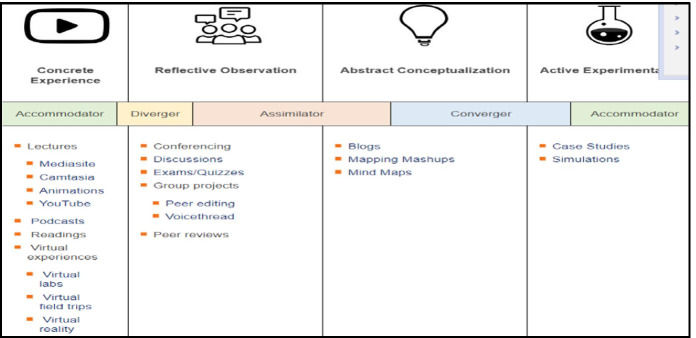
Technology Supported Learning with Kolb’s Learning Style (Source: CIIT, 2020)

These findings help identify the relevant Technology Supported Learning tools that would be capable of enhancing engineering students’ learning styles in order to increase their learning competencies within the experiential learning process. In support of this, a study by (Solvie & Kloek, 2007) aligned the Technology Supported Learning with each stage of Kolb’s learning cycle and the associated competencies. Interestingly, the ‘converger’ and ‘accommodator’ learning styles can benefit from a number of TSL tools such as virtual labs, virtual reality, simulations, u-tubes podcasts animations, etc. as shown in Fig. 2.

These important findings from literature will therefore help guide this study while evaluating the students’ experience due to the online migration amid COVID-19 and to whether the TSL tools used were sufficiently effective to enhance the engineering students’ learning experience based on the selected case study.

### Technology supported learning during COVID-19 pandemic

**Fig. 3:**
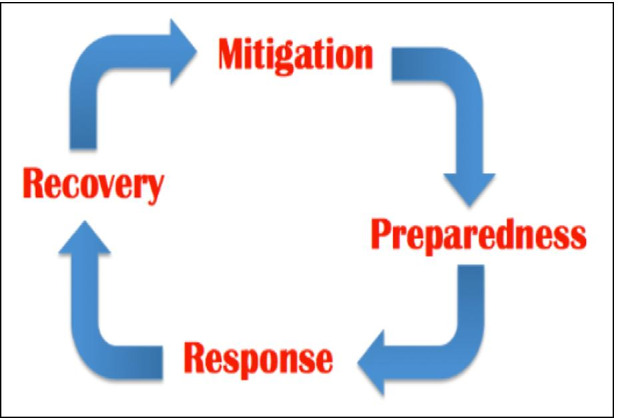
Emergency Management Life Cycle. (Adapted from Cutter, 2003)

This study argues that despite the availability of the technology that could mitigate the arrival of an emergency crises such a Covid-19, the academic communities around the world were not were prepared to deal with such crises. As such, this paper is viewed through the lens of the Emergency Management Life Cycle theory focusing on the ‘Preparedness’ and ‘Response’ phases of the four-phase model which includes mitigation, preparedness, response, and recovery as shown in Fig. 3. According to Cutter (2003), during the ‘Preparedness’ phase, actions are taken prior to an emergency to facilitate response and readiness whiles the ‘Response’ phase describes the actions taken during the emergence to reduce the negative impacts. Actions are taken after the emergency to restore normal operations and services during the ‘Recovery’ phase. At the ‘Mitigation’ stage, efforts to reduce the effects or risk associated with the hazard are deployed (Cutter, 2003; Han et al., 2012; National Governors Association, 1979). Globally, educational institutions faced many challenges in delivering teaching and learning during the COVID-19 pandemic in the adoption of technology supported learning. Sawalha (2020) argues that emergency management rely heavily on planning and therefore the ‘Preparedness’ phase is the primary cornerstone in the emergency management cycle. It is however known that, people respond differently during emergency irrespective of the level of general preparedness.

Even though the adoption of Technology in learning has increased over the past two decades, the outbreak of the COVID-19 pandemic was responded to by the closure of schools and higher education establishments globally. In support of this, Giroux (2020) argued that COVID-19 pandemic is more than a medical crisis as it continues to inflict chaos and confusion in the global education system. As a result of the COVID-19 pandemic, in-person instruction was suspended and replaced with remote teaching technologies with billions of students/pupils taken out of the classroom due to the closure of schools. Although the move of teaching and learning online enables flexible delivery anytime and anywhere, temporary move of teaching and learning to an alternate delivery mode due to the crisis (Emergency Remote Teaching-ERT) is aimed at setting up a quick and reliable learning environment in place of the normal face–to–face teaching (Hodges et al., 2020).

Technology supported learning is therefore critical for remote learning as the COVID-19 pandemic continues and adopting the use of Technology in teaching and learning is the only way to reduce the negative impact of the educational disruption caused by the COVID-19 pandemic. In addition, Scully et al. (2021) argue that technology supported teaching and learning became the only option in 2020 as a result of COVID-19 while the online learning provided during the pandemic was challenging and affected the delivery of quality teaching and learning. In addition, technology supported learning is likely to be placed at the centre of educational delivery as hybrid online approaches become the new norm for now and the future. Doucet et al. (2020) add that blended learning pedagogy that combines online, classroom and face to face teaching and learning will be the new norm in the education sector post-COVID. While remote teaching, online and distance learning are not new approaches to pedagogy or curriculum design, these are becoming more important (Williamson et al., 2020) as the current and the future education systems around the world are becoming increasingly platform-based (Hillman et al., 2020). As the higher education sector move to the adoption of remote delivery of teaching and learning in response to the current COVID-19 crisis, it is important to know that student learning experience from a well-planned online learning will not be same as the current panic response to the COVID pandemic (Hodges et al., 2020).

As the COVID-19 causes disruption at all levels of education, Doucet et al. (2020) argue that the approach should be ‘Maslow before Bloom’; this means that safety and wellbeing must come first at all times before teaching and learning as students and academics face mental, emotional and physical challenges. It is believed that the Maslow’s hierarchy of needs should be taken care of first (students safety and basic needs) before academics proceed to teaching and learning (Doucet et al., 2020). Yates et al. (2020) used a mixed method approach using qualitative data to investigate the relationship between technology and pedagogy for learning during the COVID-19 lockdown. On the other hand, Kearney et al. (2012) developed a framework that is underpinned by socio-cultural perspective and provides three pedagogical characteristics (personalisation, authenticity and collaboration) that impact on student learning experience. This research also examines student learning experience of digital learning at home during COVID-19 pandemic through the lens of Kearney et al.’s framework (2012).

The COVID-19 pandemic means that academics are facing challenges requiring the need to shift teaching online using digital tools that means new approaches to teaching and learning have to be developed since some of the face–to–face pedagogically approaches may be inadequate (König et al., 2020). While the transition to online teaching and learning in response to the COVID-19 pandemic was rapid and unprecedented creating many challenges for both individual academics and students (Ferdig et al., 2020; Howard et al., 2020) due the need for special technological skills and different pedagogical approaches to deliver online teaching and learning (Gurley, 2018; Howard et al., 2020), unfortunately, many academics and academic institutions’ readiness to transition to online teaching has been questioned (Howard et al., 2020). This is mainly due to a number of challenges that are likely to have presented themselves. Such challenges are often presented by cited by literate and fall into three main categories; ***Technological, Pedagogical and Psychological***. The technological challenges for example are faced by familiarities with teaching platforms and tool, access for technical support, as well as speed and connectivity issues (Heyman, 2010; Brittany, 2015 and Fedynich et al., 2015). Whereas the pedagogical challenges evolve around the access to teaching material, active engagement in learning, assessments and feedback and teaching styles (Abhinandan, 2020; Boling et al., 2012; Brittany, 2015; Fedynich et al., 2015; Heyman, 2010). The psychological challenges however are often associated with the students’ degree of engagement and interaction with the instructors or other students, coping with frustrations and anxieties as well as time management (Balil & Liu, 2018; Brittany, 2015; Chiu-Lan & Ming, 2020; Dhawan, 2020; Fedynich et al., 2015). However, Teräs et al. (2020) argue that educational institutions adapted quickly adapt to the COVID-19 situation by pushing teaching and learning online to ensure that students can still receive the needed education despite the crisis. Starkey (2020) argues that technology supported learning develops digital literacy, critical thinking and collaboration that enhances students’ learning experience. According to Yates et al. (2020) effective use of technology supported pedagogy enhances and motivates collaborative learning activities that improve student learning experience. To ensure efficient teaching and learning, ‘Engineering Education’ as a specific discipline of the higher education is particularly described as unique in its teaching and learning; using practical exercises, experiments and laboratory methods (Ożadowicz, 2020). Teachers/academic adopted both synchronous and asynchronous approaches to delivering teaching and learning that provides interactive and collaborative activities in response to the COVID-19 pandemic to support students’ learning (Starkey et al., 2021). As the World Bank describes the COVID-19 pandemic is a stress test for the global education system, as educational institutions are moving teaching and learning online on a scale never seen before due to the COVID-19 pandemic.

It is believed that COVID-19 pandemic will have lasting impact on the higher education sector as there is an increasing doubt about the return of global education to normality any time soon. For this reason it is important to take forward the lessons learnt from the sudden migration to online delivery in order to provide robust solutions for future education. This study will therefore shed light on the current practices in respect to the challenges and opportunities faced by academics and students amid Covid-19 based on selected case-study for a College of Engineering at a Higher Education Intuition in the UAE as described below.

### A selective case study

This research focuses on the College of Engineering at a Higher Education Institution in the UAE as a case study to identify the drivers and challenges faced in their Teaching and Learning practices following the sudden online migration amid COVID-19. The selected case study is an independent non-profit educational institute, based in the United Arab Emirates (UAE), and is recognized for its distinctive teaching, learning, research, scholarships, educating, and mentoring of future leaders. It is ranked first (#1) in the world in terms of diversity, with an 88% employability rate, while ranked within the world’s top 50 universities under 50 years of age by QS world ranking, and is amongst top 10 in the Arab world for the past 5 years with 66% students awarded scholarships. The university enrols a number of diverse nationalities with 18% Locals, 13% Jordanian, and 12% Asians as the top nationals. In addition, in the year 2019, the university has a good balance of 52.8% female to 47.2% male. The college of Engineering is the largest of the four colleges in the University with more than 2300 registered engineering students, amongst which, 68% are Undergraduate students, 13% are MSc students and 1% PhD students.

In September 2019 the college of Engineering moved into its new sustainable building for which it gained a Pearl rating. The building is equipped with the latest state of the art smart technologies including with Blackboard iLearn as a learning management system, Panapto and lecture capture facilities with built in cameras in classrooms, interactive smart TVs and censored lighting. Although the college of Engineering was ‘well’ equipped with the technology had taken small steps toward a more blended learning approach, it was not licensed to use any form of online teaching while awaiting the ministry of education approval. Therefore, in the absence of an e-learning strategy in place, the arrival of COVID-19 took the entire institution, including the college of Engineering by surprise. Nevertheless, the college excelled in coming up to speed with their Technology Supported Learning Training program to cope with the circumstances surrounding COVID-19 with strict instructions to meet the following deadlines;

- 2nd March 2020, all faculty must complete IT training for online delivery within 3 days
- 6th March 2020, Students must study from home and faculty deliver online
- 19th March 2020, Faculty work from home

With 97 working faculty and 6 Engineering departments (Electrical, Mechanical, Industrial, Chemical, Civil and Computers Science and Engineering) this was a big ask. However, despite all the challenges and unlike some universities across the world, the education process at the selected case study was not interrupted and all lectures were smoothly transformed online within the set deadlines. This paper will therefore look into the challenges faced by faculty and students through the online transition period and the coping strategies and lessons learnt for more effective delivery of future online education.

## Methodological steps

This section defines the methodological steps followed in order to explore the reaction of both students and faculty towards the sudden migration online amid Covid-19, the perceived good practices adopted by instructors and the impact of the associated challenges on the students’ learning cycle while delivering online teaching in a state of emergency. These steps entailed collecting both qualitative and quantitative data using the College of Engineering at the selected UAE Higher Education Institution as a case study. Below are the methodological steps followed in this study;

***Students’ survey***: An online survey consisting of three main parts was conducted targeting engineering students. The first part of the survey aimed at quantitatively evaluating the students’ initial reactions towards the sudden migration online, while the second part looked to identify the students’ preferred course instructor based on their online experience amid Covid-19 and the third part of the survey sought the participants’ comments on the reasons behind their choice of their preferred instructor. To aid this study, a thematic analysis of students’ perceptions of the best online practices adopted by their preferred is produced and the most factors with a positive impact on the online experiences amid Covid-19 state of emergency is reported.

***Students’ interviews***: As set of interviews were conducted guided by the findings while targeting engineering students (from each engineering department) who engaged in the courses taught by the most voted for instructors. The purposes of the interviews were to gain a deeper insight into the students’ perceptions of good practices adopted by their favoured instructors and the associated challenges in relation to the online delivery amid Covid-19 state of emergency as well their reflections and recommendations for future delivery. A thematic analysis of the results is produced underpinned by the identified challenges and good practices, along with the set of recommendations put forward by the interviews.

***Instructor’s interviews***: These interviews were conducted by targeting the most voted for instructors reported by the students’ surveys. The purpose of these interviews were to gain a deeper insight into the immediate reaction of the instructors towards the sudden migration online, the teaching, learning and assessment challenges encountered and the associated coping strategies adopted due to the sudden online migration. The interviews also sought the instructors’ reflection and recommendations as result of their teaching experiences amid Covid-19 state of emergency. A thematic analysis of the results is conducted to underpin the most presented challenges faced by the instructors and good practices adopted to overcome these challenges, including the assessment strategies.

The findings of the study intend to underpin any impediments in students’ learning process caused by the challenges facing the online delivery, while guided by Kolb’s learning cycle and how such impediments could be mitigated through the lens of the emergence management cycle. All surveys and interviews were administered online, following all the ethical consideration and approvals set by the Institutions’ Research Board.

## Data collection and results analysis

This section reports on the data collection methods and analysis obtained from the students and faculty of the College of Engineering at selected case study in order to explore the best practices and challenges of online delivery perceived by both groups.

### Students’ survey

An online survey was posted towards the end of the spring semester in 2020 targeting the Engineering students at both UG and Graduate levels, while engaging in their programs of study remotely and for the first time amid Covid-19. The survey was designed in three parts to understand the students’ initial reactions to the shift to online delivery amid COVID-19 and identify their perceived online best practices as experienced by their favoured instructor, in addition to the challenges faced and reflections upon their online experience. As such, this section shared the results of the students’ survey which is formed of three main parts;

- Part I: Captures the students’ initial reactions towards the sudden migration online.
- Part II: Determines the students’ preferred course instructor based on their online experience amid Covid-19.
- Part III: Seeks the participants’ justification for reasons behind their choice of their preferred instructor.

As such, the student population at the College of engineering at the selected case study was targeted and a total of 297 responses were returned, which is approximately 13% of its Engineering population and statistically is deemed to be a good representation of the college of Engineering population. Amongst this percentage, 98% of the respondents are undergraduate students, and 2% graduate students. This representation is a reflection of the college of engineering population described earlier in the case study section.

**Fig. 4:**
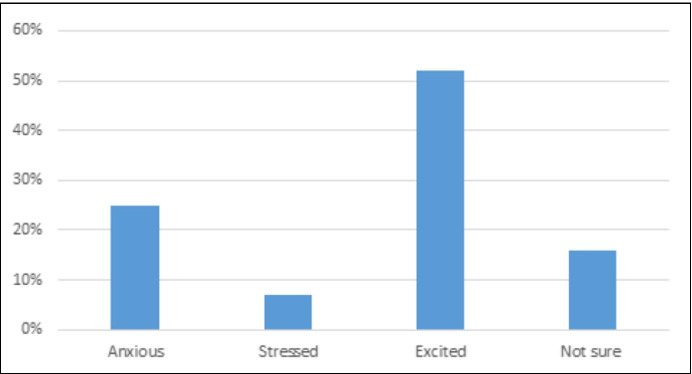
Students’ reaction towards the sudden online migration

*Part I*: To evaluate their ***initial reaction**** towards* the sudden migration online, the student were asked to select one of the following options to describe how they felt at that stage; *Excited, Stressed, Anxious, Not sure.* Figure 4 shows the breakdown of the percentages of students’ who expressed their initial reaction towards the sudden migration online.

The results showed that the students’ immediate reaction was mixed. Whereby, 52% of the respondents felt excited about the sudden online migrations, while almost quarter of the respondents either felt stressed or anxious, and a quarter of the respondents were not really sure what to think of the online migration. There is no doubt that these reactions were likely to provoke different reactions from their instructors, which will be discussed later.

**Fig. 5:**
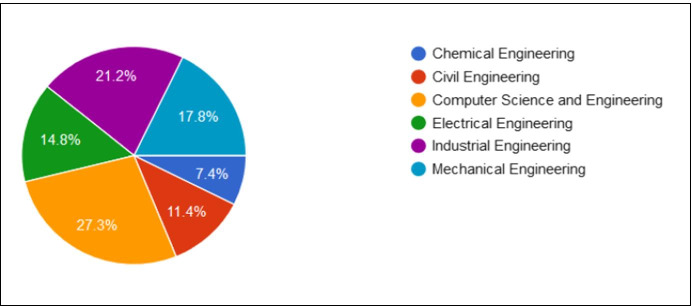
Percentage of responding students from the different engineering departments at the selected case study. (Source: Authors)

*Part II*: The second part of the survey intended to gain a deeper insight into the students’ preferred online teaching practices, while requesting them to identify their preferred instructor and portray their perceptions of good teaching practices that were introduced to time by their preferred instructor. Out of the 297 retuned responses, 69 instructors were nominated from a list of 97 instructors who worked at the college of Engineering. Figure 5 shows a fairly balanced percentage of students who responded from the six engineering departments, while the analysis of the results showed a broad range of students’ nominations [between 1 and 23] with a maximum of 23 votes gathered for one of the instructors. Students who wished to nominate more than one instructor, they were requested to fill in a new online survey.

*Part III*: In this part, the surveyed students were asked to give their reasons for nominating a particular instructor. The students’ qualitative responses were analysed and grouped into three main categories; Pedagogical reasons, Psychological reasons and Technological reasons.

**Fig. 6:**
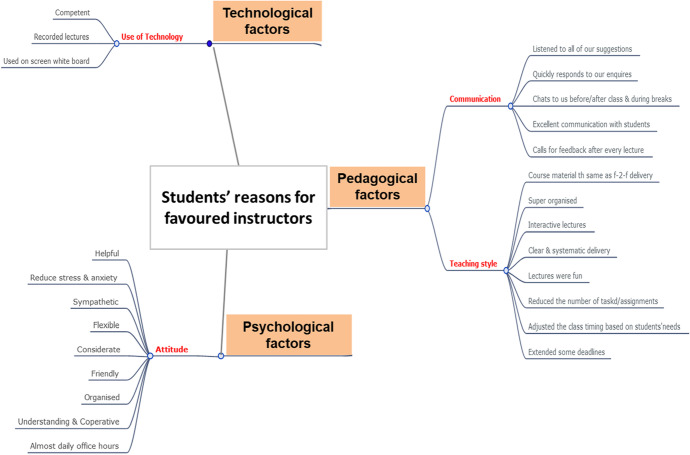
Reasons for preferred instructors. (Source: Authors)

As such, the factors that evolved around the instructors’ *teaching styles were considered ****as pedagogical factors***, which included responses such as; the efforts the instructors put into *organizing the course material, managing the class time, developing assigned tasks, making the sessions interactive and ensuring that the students achieve the same learning outcomes as the f–2–f delivery. In addition this category included factors that related to the instructors’ communication style* such as; the *intensity of advice received*, conscious efforts put into *talking to the students*, *timely feedback* etc.… For example, one the respondents stated that “The *instructor hangs around before and after the lectures, constantly listens to our needs and gives feedback to the students*”. Another student note “*The instructor ask for feedback after each lesson and address our concerns immediately*”. As for the ***technological factors*** these included praises for instructors who were ‘*competent users of the technology*’ or ‘*being creative and innovative with the use of different tools to engage the students online*’. One of the tools that was mentioned and particularly liked by some of the respondents was the use of *‘whiteboard within Blackboard Collaborate*’ which mimics the use of the whiteboard in f*–*2*–*f lectures, giving the students the opportunity to also write on the board. The students were also appreciative of instructors who recorded their *‘live lectures’* and made them accessible to the students. As for the ***psychological factors***, these seemed to be mainly related to the instructors’ *attitude* such as their *supportive behaviour, flexibility, friendliness, shared sympathy towards the students, understanding and accommodating students’ needs.* Figure 6 shares a summary of these findings.

**Fig. 7:**
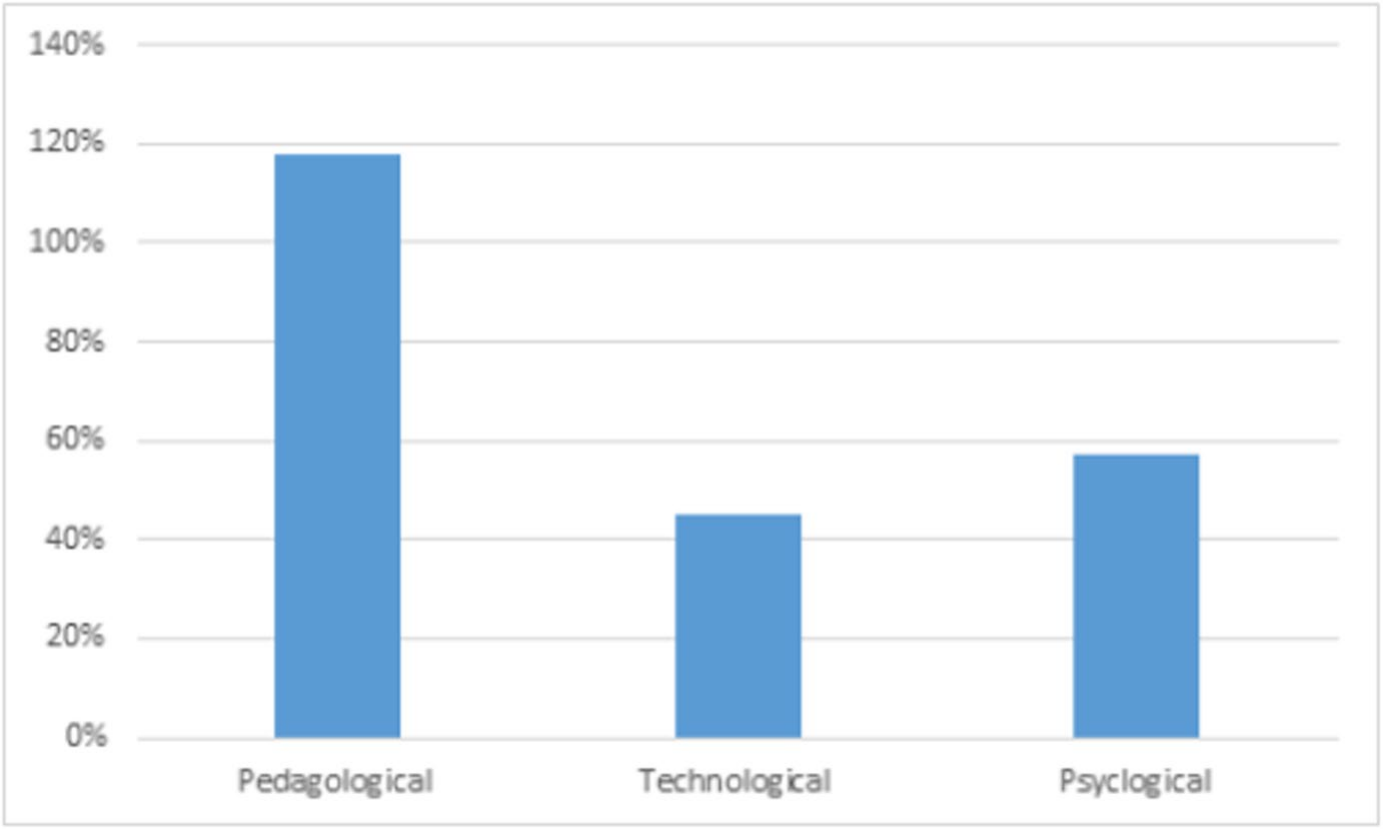
Percentages of number of comment on reasons for preferred instructors

The content of the responses were further analysed and the frequency of their appearance under each category were counted. Figure 7 shows the percentages of the frequently quoted under each category, with the pedagogical being the most quoted category for favouring the instructors. This was followed by the psychological factors and finally the use of the technology.

It can therefore be concluded that the ***pedagogical factors*** were amongst the most responses that influenced the students’ choices of their preferred instructor through the online delivery amid COVID-19, this was followed by the psychological factors based on creating an engaging environment for the students and listening to the concerns and needs, which is the resultant of the instructors’ attitude and finally the technological factors that evolve around the instructors’ competence and use of technology.

### Students’ interviews

To gain a deeper insight into the students’ preferred online teaching practices and the associated challenges as a result of the sudden online migration amid-Covid-19, thirteen students were interviewed. The selected students were identified from the courses taught by their nominated instructor, across all engineering departments (6 Industrial Engineering, 3 Chemical engineering and 4 Computer Science and Engineering students) at the college of engineering of the selected case study. Three of these students were at studying at graduate levels and 10 at UG levels. The interviews were conducted online and lasted around 25 min each, while all ethical issues were communicated with the participants. Guided by the students’ survey results, the participants were asked to;

- Explain how they felt since the online migration which took place few week earlier.
- Highlight any technological and pedagogical good practices and associated challenges and any psychological factors that may that may have impacted on their learning process.
- Project their Reflections and propose recommendations.

In response to ***Initial reactions to the shift to online delivery amid COVID-19 and how they felt since the online migration which took place few week earlier***, the students gave mixed opinions. All three graduate students stated that initially they felt excited about the online migration and remote teaching because this suited their working life style and having to travel long distances in order to join their evening classes at the university. They also confirmed that they preferred the online delivery, although they missed working with their fellow classmates on group projects while on campus. Three of the UG students felt that initially the idea of online learning seemed exciting to them, but afterwards the excitement reduced as they felt the campus life was more fun and they preferred attending their classes on campus. Five of the UG students initially were either anxious or not sure of the new remote mode of delivery, but later on felt it was manageable and enjoyable, although they missed out on the social aspects of campus life. Four of the students felt initially stressed because they were not equipped with the relevant IT gadgets such as personal laptops, mics and cameras, while 4 of the students felt stressed because of the weak internet reception where they lived. However, most of the interviewees felt that they manage to acclimatise with the new situation after two weeks of the remote teaching and were in agreement that they received the right level of institutional support in terms of training and advice from IT services and their tutors.

*In response to the ****technological**** and ****pedagogical**** good practices and associated ****psychological**** or other factors that may that may have impacted on their learning process,* the interviewees gave mixed responses. In terms of the ***pedagogical factors****,* the results revealed that the students were in favour of the recorded lectures provided by the instructors and the use of online office hours. In support of this, one of the students stated that “Recorded lectures and online office hours were the best parts of this online experience, and students would even prefer to see these implemented when on-campus classes resume”. The students also noted that Kahoot, visual studio and group activities were successful online tools as these tools allow them to reflect on their learning and apply their knowledge of the learnt concepts through interacting and gaining feedback. One of the students pointed out “Kahoot was a fun and interactive application and engaging”. Using Zoom or google meet as a backup was also perceived as successful tools to support the continuity of the learning process. As for the technological challenges, these were mainly related to the speed of the internet connection and software downloads which seemed quite frustrating and had an impact on the continuity of the learning process. It is however good to know that some backup solutions were provided to support the students. This has also generated divided opinion about Blackboard collaborate as being a good and engaging platform on one hand and being slow and occasionally unresponsive on the other hand.

In terms of the ***Pedagogical factors***, the results showed that the students are generally in favour of open ended and flexible assessment methods such as project based assessments and open book exams as opposed to strict multiple choice questions under set exam conditions. On the other hand the students highlighted a number of challenges associated with the online assessments and their fairness. For example, one participants stated that “Certain professors chose to deliver math-related assessments as purely Multiple Choice Questions (MCQs) which many of us found to be unfair as it did not accurately reflect the students’ knowledge and gave them no partial credit”. As for submitting the assessment answer sheets, students found that scanning answer sheets is good for posting their answers than typing their responses, given the mathematical nature of some of the engineering subjects which are loaded with mathematical equations that are hard and time consuming to type. This also triggered the students’ dissatisfaction with copying and pasting code into iLearn textboxes, when using online multiple choice assessment questions. Students also reported their dissatisfaction with the lockdown browser and the problems associated with it. However, it seems that instructors, who mastered the use of the lockdown browser, were comfortable with it and so were their students. On the issue of fairness, students considered the use of cameras to combat cheating as a good practice and in fact are critical to combat cheating and ensure fairness. At the same time, they were unsatisfied with some of the instructors who have ‘Significantly increased exam difficulty and length to combat cheating’.

Other challenges were pointed out by the participants which may have had a *psychological* impact on the students’ learning experience, evolved around students’ engagement as stated by one of the students “it is difficult to maintain focus during the sessions without any interactions”. The students therefore recommended the use of more engaging tools such as the use of Polls during the lectures and the in class online discussions. Another challenge was the lack of recorded lecture stating “Recorded lectures were a great help for students to keep up with the pace of the material and have more time to understand and take notes from the material” another student stated. On the other hand, a student argued that “while recorded lectures serve as a supplement to help students fill in the gaps in the material that they have missed. Professors should instead resort to more proactive ways of keeping students engaged such as polls or in-class discussions”. Therefore, both are needed, the recorded lectures and students’ active engagement. In addition to this, some students felt that there were no interactions between the students during the lectures stating “in my class there was virtually no student–to–student interaction in Blackboard Collaborate. Nonetheless, other means such as WhatsApp groups are available”. Therefore, it is important to create forums for students’ interaction within the lecture sessions; this could be topped up with group assignment tasks that would create a less isolating environment for students.

Some of the students also highlighted the challenges facing the smooth delivery of lectures due to the ineffective use of the technology as mentioned by one of the students “In-class communication was slower than usual due to the need for the professor to close/re-open the chat, open/close mic for students, etc.” This issue, coupled with the instructors’ ability to cope with the technology, called for the students to recommend for instructors to be well trained to deal with the smooth running of the sessions. Although this study revealed that backup platforms were used when Blackboard Collaborates was slow in its response, some of the students seem to have been missing this option and recommended the use of other platforms such as Zoom, and Google meet as a backup.

The summary of the interview findings can be seen in Table 1 whereby the factors that have supported the students’ online learning and their associated challenges have been presented in three categories (technological, pedagogical and psychological.

**Table 1:** Factors and challenges associated with students’ online learning amid Covid-19

	Good practices	Challenges
Technological	•Blackboard collaborate is a good and engaging online platform •Recorded Lectures helps students keep up with pace of online learning and make up for its limitations •Visual Studio Live which is a programming platform, that enables students and instructors to interact with the same code online in real time •Zoom or google meet used as a successful alternative to Blackboard Collaborate to make up for its limitations •Online Office Hours were found very helpful and convenient by students •Kahoot—Interactive quiz application to keep students engaged	•Connectivity issues and slow software downloads through remote access. •Blackboard Collaborate slow in terms of connectivity and real time interaction •Slow in-class communication slower than usual due to close/re-open the chat, open/close mic for students, etc. •Inability of some professors to adapt to the online teaching environment and tools
Pedagogical	•Utilisation of Project-based assessments instead of timed online exams •Open-book assessments that shifted focus from memorizing concepts to proper and efficient application •Scanning sheets of paper (containing solutions) and uploading to iLearn; regarded as “best method” by students •Use of Webcams (to combat cheating) with exams of reasonable length and difficulty •Utilising Group activities	•Blackboard Collaborate found to be adequate and sufficient to mimic the class-room environment, but some students. •Some lecturers were not competent with the use of the online tools which wasted some of the lecture time •Lack or no students-2-students interactions within the online classes •Difficulties to stay focused without class interactions. •Inability of some professors to adapt to the online teaching environment and tools. •Working in isolation •Pure multiple choice assessment questions (especially on mathematics-based courses) •Significantly increasing exam difficulty and length to combat cheating •No-backtracking policy to combat cheating •Copying and pasting code into iLearn textboxes (for programming courses) not suitable for engineering courses •Practical assessments at home should be given more time and/or more assistance to account for technical issues •Lockdown Browser gave rise to many technical issues such as mathematical formulas formatting, starting up, etc. •Limited or no opportunities for class interactions. •Absence of recorded lectures for certain courses. •Group Activities promoted student–to–student interaction while providing additional engagement for students which was absent in Blackboard Collaborate lectures
Psychological	•Working in groups •Interactive lectures •Having recorded lectures and supportive material as additional resources •Ensuring faire assessments procedures and formats •Smooth online delivery •Having access to the instructor for support and feedback	•Difficulties to stay focused without class interactions. •Inability of some professors to adapt to the online teaching environment and tools. •Working in isolation •Slower in-class communications. •No student–to–student interaction in Blackboard Collaborate. •Connectivity issues in Blackboard Collaborate •Absence of recorded lectures for certain courses

#### Students’ reflections and recommendations

The students were asked to highlight any challenges and recommended solutions upon their reflections of their online migration experience amid COVID-19. The results showed that the highlighted challenge (See Table 1) evolved around students’ engagement as stated by one of the students “*it is difficult to maintain focus during the sessions without any interactions*”. The students therefore recommended the use of more engaging tools such as the use of Polls during the lectures and the in class online discussions. In support of this one of the students mentioned. Another challenge was the lack of recorded lecture stating “*Recorded lectures were a great help for students to keep up with the pace of the material and have more time to understand and take notes from the material*” another student stated. On the other hand, a student argued that “while *recorded lectures serve as a supplement to help students fill in the gaps in the material that they have missed. Professors should instead resort to more proactive ways of keeping students engaged such as polls or in-class discussions”.* Therefore, both are needed, the recorded lectures and students’ active engagement. In addition to this, some students felt that there were no interactions between the students during the lectures stating “*in my class there was virtually no student–to–student interaction in Blackboard Collaborate. Nonetheless, other means such as WhatsApp groups are available*”. Therefore, it is important to create forums for students’ interaction within the lecture sessions; this could be topped up with group assignment tasks that would create a less isolating environment for students.

Some of the students also highlighted the challenges facing the smooth delivery of lectures due to the ineffective use of the technology as mentioned by one of the students “*In-class communication was slower than usual due to the need for the professor to close/re-open the chat, open/close mic for students, *etc*.*” This issue, coupled with the instructors’ ability to cope with the technology, called for the students to recommend for instructors to be well trained to deal with the smooth running of the sessions. Although this study revealed that backup platforms were used when Blackboard Collaborates was slow in its response, some of the students seem to have been missing this option and recommended the use of other platforms such as Zoom, and Google meet as a backup.

Although this study revealed a number of positive technological, pedagogical and psychological strategies were used introduced by some of the instructors, the associated challenges could have played a role in hurdling the students’ experience. For example, the slow connectively and download issues, coupled with the absence of recorded lectures in addition to the instructors’ inconstant use of the technology would easily deter the 1st stage of Kolb’s learning cycle and that is engaging in a meaningful ‘experience’. In addition, inefficient and/or ineffective formative or summative assessment strategies, would deter the 2nd stage of the learning cycle, which is reviewing and reflecting upon the given experience). When this happens there is no doubt that this will have an impact on the 3rd and 4th stages of the learning cycle (abstract conceptualisation and active experimentation), which results in students’ passive participation engagement in class. It can therefore be argued that the lack of technological and pedagogical preparedness amid the state of emergency presented by Covid-19, presented the students an appropriate response which has been cushioned (mitigated for) through the psychological support given by caring instructors through a flexible and responsive attitude. However, to ensure an effective learning process, more preparations are required in terms of the instructors’ training, the provision of solution for slow connectivity, better assessment strategies and the integration of more interactive learning. As such, the participants proposed a set of recommendations that could help instructors mitigate for future online delivery as stated by one of the student argued that “*Emphasis should on open discussion during online lectures and engaging the students using different tools such as polls and in-class questions*” whole another student stated that “*Professors should therefore consider practicing and mastering the online tools….. Professors should learn to manage the online environment more efficiently”* finally *“Professors* should create forums for students’ interaction within the lecture sessions. It is therefore important to look into the emergency management cycle to see how the present of the impediments that deterred learning can be mitigated for to ensure better preparedness for future online delivery.

Finally, when synchronous and asynchronous modes of delivery are used to allow students engage effectively through a well-structured use of technology that mimics effective f*–*2*–*f teaching and supports the different stages of the learning process allowing students’ engagement and interactions to reinforce the leant concepts, as well as having recorded lectures that allow further points of reflection, the learning process can be successfully accomplished. By overcoming some of the technical challenges such as slow internet connection and interruptions, topped with the lessons learnt from the sudden migration to online delivery amid COVID-19, will take the Engineering discipline into a new era while creating new opportunities for blended learning approaches to meet the needs of the new generation of engineering students.

### Instructors’ interview

This section shares the interview results which were conducted with the top nominated instructors by the students from each department within the College of Engineering. As a result, 15 instructors were interviewed online via ‘google meet’ for about 30–45 min, towards the end of the 2020 spring semester, around 8 weeks after the online migration. All ethical approvals were obtained from the university’s Institutional Research Board, and all ethical consideration were followed to protect the identity of the instructors by assigning a code for each interviewees, while permissions were sought for recording the interviews. Table 2 shows the profile of the interviewees. The interviews were aimed at understanding the;

- Instructors’ initial Reactions and response to the shift to online delivery amid COVID-19.
- Challenges Encountered & Solutions Employed in response to online mode of delivery.
- Assessment strategies and associated challenges.
- Reflections and proposed recommendations for other Instructors.

**Table 2:** Profile of Instructors Interviewed

Department		Gender	Position	Code
Chemical engineering		Male	Professor	CHE[1]
Chemical engineering		Female	Associate Professor	CHE[2]
Computer science and engineering		Female	Associate Professor	SCE [1]
Computer science and engineering		Female	Lab Instructor	SCE [2]
Computer science and engineering		Female	Lab Instructor	SCE [3]
Electrical engineering		Male	Professor	ELE [1]
Electrical engineering		Male	Associate Professor	ELE [2]
Electrical engineering		Male	Professor	ELE [3]
Industrial engineering		Male	Associate Professor	INE [1]
Industrial engineering		Male	Associate Professor	INE [2]
Mechanical engineering		Male	Associate Professor	MCE [1]
Mechanical engineering		Male	Associate Professor	MCE [2]
Civil engineering		Male	Professor	CVE [1]

The rest of this section presents a summary of the thematic analysis of the main findings.

#### Initial reactions and response to the shift to online delivery amid COVID-19

Given that the college of Engineering had not utilized much of Technology Supported Learning practices within its curricula before the COVID-19 crises, the purpose of this question was to understand the immediate reactions and response of the course instructors towards the sudden need for online delivery, and their views of the students’ reaction and response to the sudden online migration. The interview results showed that the instructors’ reactions and their immediate responses were generally aligned. For example, CHE [2] and ELE [2] response evolved around the need to understand ***how the technology works***. *CHE [2]* noted that her immediate reaction was recognizing the need that she must be in control of the use of technology and that all her students must be trained to use iLearn collaborate before they studied at a distance. While *ELE [2]* was initial reaction was concerns with about the medium of delivery, how he would be delivering his lectures, would it be live lectures or pre-recorded and how he would deal with that? *SCE [1] however,* felt a sense of excitement and an opportunity for learn something new, while INE[1] and MCE{1] and CVE [1] felt quite comfortable with the technology and their immediate reaction were mainly concerns that focused on the students and to whether they would like the online mode of delivery or not.

*On the other hand, CHE [1], SCE {2], INE [1]* initial reactions were more to do with how they will be ***Engaging and Interacting with the students**** and* to whether they would be able to cover the material adequately. For example *CHE[1] mentioned* “ *I have a highly social approach to teaching and was worried how the transition to online learning would affect that*”, while CSE [2] was mainly concerned about how she will be engaging students in hands-on Lab work and software teaching stating “*I did not know what to expect specially that I teach in the Labs.” ELE [1]* however was mainly concerned about ***mimicking the real life classroom environment*** online, stating “*Since I prefer to write on the board rather than using slides, my main concern was how to keep what I am writing in focus, and how to refer to what is written on other parts of the board since the camera in the classroom can only focus on one section of the whiteboard.* Adding, *“I was also concerned that not being able to see the students would make it difficult for me to judge if they understand the material.”* While ELE [3] stated his concerns about the changes that he needs to make in his teaching style and teaching materials in order to deliver a similar experience online as in the classroom. Other concerns emerged about the ***Potential of technology* itself** and whether it was capable of meeting the instructors’ demands and students’ expectations. *SCE {1]* for example wondered whether the use of the tool available such as iLearn Collaborate would be applicable to the courses she was teaching, and INE [2] was concerned about connectivity issues and the availability of hardware to deliver his online teaching and to whether iLearn collaborate would be sufficient to promote the degree of interactivity needed in a real life classroom environment.

As for the ***students’ reaction*** to the online mode of delivery, CHE [1], CVE [1] and INE [1] mentioned that there was a mixed reaction by the students. While some were comfortable within their own settings, some preferred the f-2-f interaction on campus. At the same time, few were ***excited*** to go through this new experience and few were ***nervous*** about the online learning and feeling ***stressed*** about the entire situation. While SCE [2], SCE [3], ELE [1], INE [1] argued that their students were initially anxious but were relieved after a couple of sessions. Other instructors sensed a more positive reaction and enthusiasm from their students. For example, CHE [2] said “*the students’ attendance and participation was remarkable*”, while SCE [1] stated “*students generally liked distance learning and mentioned that they felt they could dedicate more time to their domestic needs instead of time spent on getting ready and travelling to campus*”. ELE [2] and ELE [3] also felt that the students were very Cooperative and gave lots of feedback in the class, while MCE [1] stated that “*my students found the online teaching better than the regular classroom*”.

Given that the online migration amid Covid-19 placed the teaching and learning community in a state of emergency, found itself generally ***ill prepared*** in order to provide an immediate ***response*** for remote delivery, although the extend of preparedness amongst the instructors varied. Hence, some instructors lacked the technical skills required to use the technology, and others lacked the pedagogical skills needed to engage the students effectively through remote delivery. There is no doubt that the instructors’ delayed responses to the students in terms of managing the educational process would have raise psychological and pedagogical concerns that are likely to have impacted upon the students’ learning experience within Kolb’s experiential learning cycle.

#### Challenges encountered and solutions employed in response to the new mode of delivery

The interview results showed that course instructors encountered a number of challenges in response to the need for remote delivery of teaching but also developed some coping strategies and solutions deployed to deal with these challenges. Based on the interviewees’ results, these challenges are grouped into three categories; technical, ethical and pedagogical challenges as discussed below and are summarized in Table 3.

**Table 3:** Summary of the challenges faced by the instructors and the solutions deployed

Challenges	Issues emerging	Solutions deployed
Technical	-Occasional internet connection interruptions -Students’ connectivity issues causing delays in joining the sessions	Recorded and archived the lectures for students’ access. Used other platforms such as ‘Google meet’ as a back platform in case of interrupted connections via iLearn collaborate
	-Displaying complex mathematical equations and derivations on the shared screen	Use digital tablets and other tools such as Microsoft whiteboard for writing on shared screens
Pedagogical	-Students’ engagement online	Verbal and periodic questioning during the lectures Active approaches to teaching by picking students at random to answer short questions Use Kahoot which is a quiz game to engage students in answering questions in a fun and competitive way Giving the students presenter rights to write on the shared digital screen Use polls in a form of multiple choice questions to evaluate students’ learning Use discussion threads in synchronous modes to encourage students’ engagement. Mark students for active participation Introduce breakout sessions on Collaborate and engage students to work in groups
	-Difficulties in using the same lecture slides as f-2-f teaching	Enrich the lecture slides with questions for the students to interact and respond to Leave gaps between the lines on the slides to write on
Psychological	-Dealing with Students’ anxieties and uncertainties -Students’ isolation and lack of peer interactions	Increased office hours to give more attention to the students Allow the students to undo their feelings during the lecture session

***Technical challenges***: The interviews referred to a number of technical challenges faced during their teaching semester online, these included;

- ***Internet connectivity issues***: Although bandwidth limitations and their impact on the internet connectivity was not a nagging problem that faced the online delivery at selected higher education institution, there were few occasions at the start of the online migration period when the internet connection was interrupted by the service providers to cope with the sudden surge of internet demands across the world. At the same time, students would have bad connectivity, causing them to leave the online sessions with attempts to re-join again. To overcome such interruption issues faced during the synchronous mode of delivery, ELE [1] and CHE 2] reported that they used recorded their lectures via Blackboard Collaborate and archived them on Blackboard iLearn for students to listen to them in case they missed out on the material delivered. While CSE [1], MCE [1] used other platforms such as ‘Google meet’ as a back platform in case of interrupted connections via iLearn collaborate.
- ***Limitation of iLearn Collaborate whiteboard and difficulties with writing equations and derivations*: **Given the mathematical nature of some engineering subjects, this requires the display of complex mathematical equations and derivations, this issue was perceived as a challenge to some of the instructors. However, CHE [2] mentioned that he overcame this challenge through the use of digital tables and other tools which allowed for onscreen free handwriting. ELE [2] and INE [2] also found teaching mathematical concepts online challenging, so they tried to learn and explore alternative tools to demonstrate mathematical concepts in addition to iLearn Collaborate and used alternative software such as Microsoft whiteboard and Microsoft OneNote. These tools allowed more flexibility and space for on screen writing.

*Pedagogical challenges*: As described by literature, the art of teaching (teaching pedagogy) requires a set of structured instructions that are mapped to the students’ needs, students’ engagement and the evaluation of students’ learning through feedback. Therefore, there were a number of challenges reported by the course instructors in relation to these challenges.

- ***Students’ engagement during the lecture*: **One of the main concerns that were regularly reported by the interviewees was determining whether students were following through the lecture online—To overcome these concerns, CHE [2], CHE [1], CSE [1], ELE [2], ELE [1], MCE [1], MCE [2] used verbal and periodic questioning and active approaches to teaching by picking students at random to answer short questions. For example, CVE [1] mentioned that extra effort was made to call out each student to ensure that they are present and engaged. CHE [2] also used Kahoot which is a quiz game to engage students in answering questions in a fun and competitive way. Blackboard discussion boards were also used by CHE [2] to post threads of discussion topics for students and instructors to engage any time asynchronously and address their discussion threads during the online sessions. While Polls in a form of multiple choice questions were used by CHE [1] and CSE [1] to evaluate students’ understanding of the lecture and keep them engaged. While MCE [1]Gave students marks for their active participation in class, for longer lab sessions, SCE [2] used breakout sessions on Collaborate and engaged students to work in groups while constantly going around to make sure they were fully engaged in the given task. As for longer lab sessions, CSE [2] randomly selected students and allowed them to share their screen to show their progress to the rest of the class. A similar approach was adopted by CHE [1] giving the students presenter rights to write on the shared digital screen. To ensure students’ full engagement and attendance, CSE [3] took screen shots of students’ attendance list at the start, middle and end of the session and marked their participation in class.
- ***Difficulties in using the same lecture slides as f-2-f teaching.*** Most of the interviews reported on their awareness of the importance of adjusting their lecture material in order to cope with the required style of delivery online. To overcome this, CME [1] enriched his lecture slides with numerous questions to keep the students engaged and allowed spaces between the lines to write on them.

*Psychological challenges*: The interviewees also reported on the psychological impact that the COVID-19 has on the students and the anxiety created due to the uncertainty of the situation and feelings of isolation from their peers and instructors. Therefore, some good efforts were reported to engage the students and ease their pressure. For example, INE [1] made sure that students were communicated to constantly by holding regular meetings and continuously seeking feedback after each session. MCE [1] allowed students to share their feelings in class about the lockdown, stating “*this kept them engaged and refreshed from the normal class rhythm*”.

**Fig. 8:**
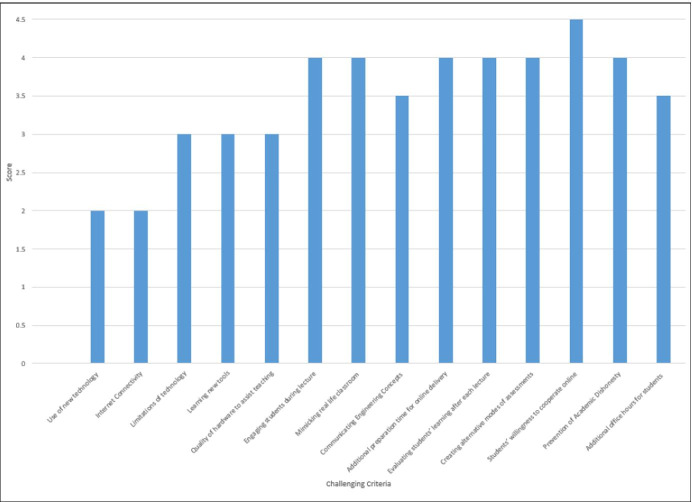
Challenges faced by Instructors during online teaching migration amid COVID-19

The pedagogical challenges encountered, were centred on the “lack of familiarity with the new teaching technology, difficulty of lab work being conducted online,” “Oversaturation [of] using online tools in the learning process” and “the lack of interaction with professors”. Faculty instructors feel that students are more susceptible to psychological challenges with regards to communicating with each other and establish their social support network, and facing problems in planning for their futures. Having identified these challenges, a short survey was conducted asking the college instructors to give a score of (1 least challenging and 5 most challenging) to the most challenging aspects of the online delivery. Figure 8 below shows the scores of 44 instructor responses out of 79 from the college of Engineering at the selected case study.

The results show that the most challenging aspects of facing instructors during the online delivery is the students’ willingness to cooperate within the online environment (with an average score of 5) with the least challenging aspect is the use of the technology. This goes to prove that the technical aspects are less concerning/challenging to the instructors when compared to the pedagogical and psychological factors. However, had the instructors been in a better state of preparedness for remote delivery during such state of emergency, they may have responded to students’ needs by engaging them more in class using a combination of technological and pedagogical strategies. According to Kolb’s experiential learning theory, failing to meet students’ in a way that matches their learning styles, will result in them dropping out of the learning process, which is what seems to be happening during this state of emergency.

#### Assessment methods

**Fig. 9:**
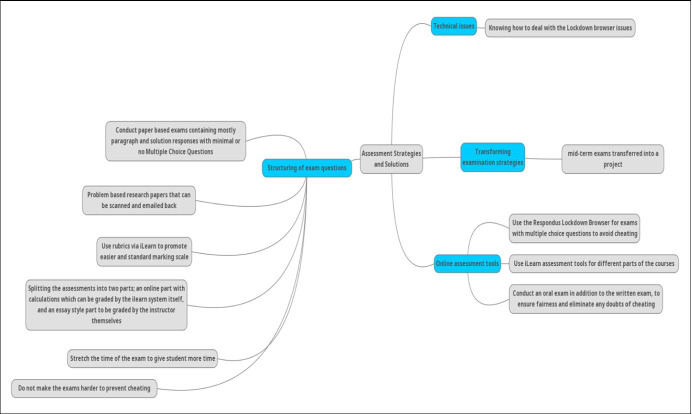
Summary of the assessment strategies adopted as a result of the online migration amid COVID-19

Prior to COVID-19, all the exams at the college of Engineering were conducted in-house on campus. Therefore, one of the major challenges that faced the college of engineering due to the sudden shift online was students’ assessments. As a result, the course instructors had to steer their thoughts and directions towards developing different strategies to ensure a practical (yet fair) way of conducting the course assessments. Some of these strategies reported by the interviewees are discussed below and shown in Fig. 9.

- ***Transforming examination strategies***: CHE [2] transformed her students’ mid-term exams into a project to better suit the online learning setup, while quizzes and homework assignments were kept the same.
- ***Structuring of exam questions***: CME [1] and CSE [1] conducted paper based exams, which mostly contained paragraph and solution responses with minimal or no Multiple Choice Questions. Assessment in a form of a problem based research paper was also imposed by CME [1], CSE [1], ELE [3], whereby the students were requested to scan and email their answers. As for marking the assignments, iLearn rubrics were used by INE [2] to imbed the assessment criteria for more consistent and ease of marking. While MCE [2] worked out a different assessment strategy by splitting the assessments into two parts; an online part with calculations which can be graded by the ilearn system itself, and an essay style part to be graded by the instructor themselves. MSCE [2] further advised that a hybrid approach was found to create discrepancy in entering grades; therefore separate questions/sections are more straightforward. CME {2} further stressed that “whatever strategy is used, instructors should not design exams under the assumption that students will try to cheat and they should assume that the students will behave fairly and professionally, therefore making exams harder is unfair to students who would not cheat”. In addition, to ensure fairness in terms of the duration of the exams, MCE [2] stated “I stretched the duration of the exams by 15 minutes more than the regular exam time to give the students’ sufficient time to think over the questions”.
- ***Online assessment tools*–** CME [1], CSE [1], CVE [1] used the ‘Respondus Lockdown Browser’ for exams to avoid cheating, while enriched with more Multiple Choice Questions for the same reason. INE [2] stated that he had a positive experience using the Lockdown browser, but also used the iLearn assessment tools for different parts of his courses. In addition, MCE [2] mentioned conducting an oral exam in addition to the written exam, to ensure fairness and eliminate any doubts of cheating.
- ***Technical issues***: CVE [1] stated that the Respondus browser would flag warning signs when students closed the browser; he has therefore decided to use iLearn assessments instead. ELE [2], MCE [2] however highlighted the challenges facing the students when using the lockdown browser such as the loss of internet connection during the exam, uploading the solutions to the lockdown browser, viewing formula sheets during the exam, getting flagged up without doing anything wrong. ELE [2] stated with confidence that “all these issues had their own obvious solutions and were dealt with”.

It therefore be seen that although the different adopted approaches seems valid in their own rights to respond to the Covid-19 crises, there does not seem to be a strategic or standard approach to handling such strategies to ensure fairness and consistency. Hence, this a reflection of lack of preparedness for the state of emergency brought about by Covid-19. At the same time, assessment and feedback are pivotal to the 2nd phase of Kolb’s learning cycle which entails reviewing and reflecting upon what has been learnt. Therefore, ineffective assessment strategies could case and impendent in the students’ learning process and would certainly have a psychological impact on students’ engagement in the learning process.

#### Reflections and proposed recommendations for other instructors

This section discusses the instructors’ proposed recommendations that could help other instructors ‘mitigate’ for their future online delivery with some thoughts and reflections upon their experience in order to provide a better response to the online delivery amid COVID-19 or similar crises;

##### Teaching strategies

The instructors gave a number of suggestions and tips based on their own experience, which could help other instructors with their teaching strategies amid their online migration as listed below’.

- ***Focus more on students’ engagement and interactions in class*—**CHE [2] CHE [1], CVE [1]. MCE [2] emphasized the importance of making classes more interactive and not just displaying the lecture slides. While, CHE2 argued that there are a number of available solutions that can help engage the students online, and they should not be perceived as backup options and they should be integrated into the online delivery. To add to this CSE [1] praised the number of tools available to engage the students in learning stating “*It’s really great if we can have a more engaging, interactive environment in online classes by using a mix of diverse tools like animated slides, polls, whiteboard, online programming, pop quizzes, these I think will make the sessions interactive and would be more engaging for the students*.” MCE [1] and CHEM [1] also recommend engaging the students by asking the students to solve questions using the virtual whiteboard, in addition to asking a lot of questions in class and having in class quizzes even if not counted towards grades. While CSE [3] highlighted the importance of making large classes in particular highly interactive.
- ***Take time to learn the provided tools and search for new ones to enhance teaching***—Most of the interviewees emphasized the importance of mastering the existing Technology Supported Learning tools for synchronous and asynchronous modes of delivery and inviting time into learning new ones CHE[1], CHE[2] and CVE[1]. In support of this, CHE [1] and INE [2] stressed the importance of constantly practicing these tools to make the learning process better. In addition, CSE [2] who is involved in Lab courses argued “*being creative by experimenting with different tools can make the Lab sessions more fun and more engaging*”. On the other hand, INE [2] added “*when experimenting with the use of technologies there could always be unexpected technological issues that happen, which sometimes frustrate students, instructors should therefore be patient and understand the students’ needs and their frustrations too”.*
- ***Constantly seek feedback from the students*—**The importance of seeking constant feedback from the students was one of the good practices followed by almost all the interviewees. For example, CSE [1] shared his experience during the online migration period saying, *“An ongoing feedback was sought from the students after each class or every week to get a better idea about their online learning experience…I listened and acted on feedback”.* CHE [1] also strongly recommends that instructors should be willing to listen to what students they have to say, so that the process of improvement follows. *While MCE [1] says “I arrived online a few minutes early before the lecture and stayed online after every lecture for a few minutes to talk to the students, motivate and engage them in decision making….I also had regular office hours to discuss any concerns and answer to any questions*”.
- ***Investing time into the preparation***: CHE [1] strongly advised the take extra time to prepare and organize the online lectures, stating “It *is just like any other task, if you put time into it you can become good teacher whether online or offline…..if you don’t prepare, the students are going to be lost, we are talking about engineers, it is not east*”. CVE [1] however advises course instructors to have a plan on how they will deliver the content, stating “*Have short but well-prepared, quality lectures and focus on the Quality rather than interrupted quantity”.* Practicing everything that is being introduced to the students is another advice given by CVE [1], whether it is an assessment task that needs to be flawless or material that requires students’ participation saying “*allow you to act as a student…..if it goes perfectly, it will also be flawless to the students*”.
- ***Share your camera as it helps students focus better*—**According to CSE [2] and MCE [1] students were generally more involved when the camera was on, and they strongly recommend instructors to encourage their students to turn on the cameras during the online sessions.
- ***Empathize with the students and try to understand their point of view***—CSE [2] felt that connecting and empathizing with the students can make them feel more relaxed especially in such a tough situation amid COVID-19. Empathizing with the students should be stretched beyond verbal communications, to include assessments tasks. For example, CVE [1] recommends that students should be given a better experience in solving and reviewing their assessment and considering extended deadlines. ELE[2] however warned of being too lenient with the students in terms of assessment deadline stating “ *while course instructors can benefit from the flexibility of online learning (such as scheduling assessments, assigning classwork, *etc*.) they also need to be wary about its costs* (e.g., students choosing to just submit everything on the last day)”, suggesting that *“Flexibility comes with responsibility*” in other words empathizing too much with the students can be disadvantageous in some respects.
- ***Share experiences with others so that all instructors can benefit***—INE [1] shared his thoughts on how COVID-19 created a great sense of community amongst academics and students at the college of engineering and emphasized the importance of knowledge sharing with others. *INE [1]* therefore advises instructors in this respect by saying *“Don’t just do your own thing but discuss with others to know ways in which you can help or receive new tips for things you could improve in your classes”.*
- ***Repeat and recap concept after every chapter, focus on quality over quantity*—**According to ELE[2] it is very important when teaching an online course to put one’s thought into it and to be organized, stressing that “*Before you start teaching the students, you need to think about how [you are going to] deliver this message today because … I discovered, and what a lot of colleagues also confirmed with me, [that] teaching online actually requires more effort than teaching in a classroom*.” In the same vein ELE [1] emphasized the importance of giving attention to quality over quantity. In support of the INE [2] argued “*It not about how much you cover but rather about the quality of teaching*”.
- ***Record lectures***—SCE [1] and CHE [2] mentioned that there is a great benefit to record the lectures as students can always revisit them, while MCE [1] recommended the use of educational videos too in order to enhance the students’ learning process.

All of the above good practices employed by the instructors played a role in supporting the different stages of the experiential learning cycle and in preparedness for a better response during states of emergency, as is the case amid Covid-19.

##### Technology supported learning tools used

The interview analysis identified a number of tools that were used during the online migration amid COVID-19 which were found to be quite effective to enhance their online delivery. These included;

- ***Digital Whiteboard Tools***—According to ELE [2] there were limitations in the ***Blackboard Collaborate Whiteboard Tool*** due to the lack of a more-precise eraser and its occasional unresponsive behaviour. Therefore, as a temporary solution **Microsoft Whiteboard** proved to be a more reliable medium than the Collaborate whiteboard tool, although there were exporting and importing issues faced. ELE [2] therefore recommends **Microsoft OneNote** which he considered to be the best whiteboard tool used so far which packs numerous whiteboard tools and organization schemes together into a reliable platform (Microsoft, 2020).
- ***Teaching 3-Dimensional Concepts*—**To explain 3D concepts to the students, ELE [2] used hand gestures to effectively visualize 3-dimensional concepts in electromagnetism (during the f-2-f delivery). However, this may not be possible or as effective in a 2D setting (while using Collaborate whiteboard tool). ***Paint 3D*** was therefore used instead of relying on 2D drawings, for students to better visualize 3D concepts.
- ***Synchronous Conferencing tools***: All course instructors used Blackboard collaborate for one 1–2–1 sessions and the online delivery of lectures and considered other web-conferencing platforms such as google meet and Zoom as a backup to Blackboard collaborate, given that they currently have fewer interactive features than Blackboard Collaborate.
- ***AB tutor v GoToAssist or TeamViewe***r—**AB Tutor is** the perfect classroom management software tool, allowing teachers to teach in networked classrooms and labs. CSE[3] believes that a remote version of a tool such as AB Tutor, which allows lab instructors to monitor students in the lab to ensure that they are paying attention and not misusing the lab computers, would be of great benefit for lab instructors in their future online sessions. This software is contrary to existing remote-access tools such as ‘*GoToAssist’* or ‘*TeamViewer’* which are designed to connect to and monitor only one device at a time.
- ***Polls*: **To make sessions more interactive, polls within iLearn were used by all the instructors to engage students in synchronous modes of delivery. While Poll everywhere using mobile apps were used by CSE [1] and CSE [2] and were highly recommended for conducting anonymous surveys and pre-designed assessment questions for synchronous interactions and described them as “*fun to use and engaging*”.
- ***Quality Hardware***: Investing in quality hardware such as headphones, mic, digital tablets etc.… was one of the points that were strongly raised by ELE [2] who stressed “In my opinion, if you really want to enhance the students’ experience, try to invest in quality hardware. For example, wireless noise cancelling headphones (instead of a wired headset) made me more comfortable and gave me the freedom to move around while delivering a lecture.
- ***Remote access to University Software***: Given that the selected Higher Education Institution for this study has allowed remote access to vast number of licensed software, which has long been part of its IT strategy and infrastructure, SCE [2], CSE [3] were able to download the needed software on their own machine as the AB tutor software to enable their engagement with their students and guide them through a step by step learning process.

All of the stated technological enhancements are considered means for enhancing the students’ learning experience and the pedagogy of learning in order to be more prepared in.

##### Reflections about online teaching experience

Upon reflection, the interviewees had a mixture of positive and negative views of their recent experience of the online migration amid COVID-19 as discussed below.

- **Positive Reflections**
  - All the instructors felt that the online experience has been a positive one with a steep learning curve.
  - Interviewees CSE [1], CHE [1], CHE [2] felt that the online delivery will add more flexibility and accessibility to the curricula delivery, especially for graduate levels. In support of this CHE2 stated *“Perhaps the university will open the floor for new opportunities regarding distance learning especially at the graduate level, as it provides a more flexible and convenient platform for these students who may need to commute in the evening”*
  - Interviewees SCE [3] and CHE [2] felt that the online mode of delivery is an advantage, not a backup solution, and it should be made good use of to support different models of delivery.
  - Most of the interviewee’s applause the efforts of IT services and their great efforts in providing the tools and training for faculty as and when needed.
  - CSE [3] felt that there was less noise and distraction during the online classes and perceived it as a positive outcome of the online delivery.
  - MCE [2], MCE [1], CSE [1] felt that the online migration amid COVID-19 can be the seed for the blended learning approached.
  - MCE[2], INE[2], ELE[2], CSE[3] perceived the online delivery of the curricula as a very effective methodology, as students seem to be more engaged online than in the f-2-f classes, while INE[2], INE[3], MCE[1], CSE[1] mentioned that shy students seem to open up more when online.
  - INE [2] and CSE [2] felt that teaching can now be flexibly delivered anytime and anywhere giving the course instructors the opportunity to attend events and conferences while delivering their teaching remotely.
- **Negative Reflections**
  - CHE [2] felt that undergraduate students would benefit more from an on-campus experience and that the extracurricular activities and students’ engagement in university life are just as important as their enjoyment in their studies.
  - MCE [1] did not favour the online mode of delivery and stated that “*It is only an exercise of making the best out of a difficult situation*”. CVE [1] also mentioned that the online delivery cannot replace f-2-f teaching, and is best left to unpreceded occasions,
  - According to CHE [2], CHE [3]. CHE [2] and CVE [1], face to face interaction with the students is missed as stated by CVE [1] “as an instructor I feel more energized when I am physically in the class….I also miss seeing the students, although I could hear their voices”. In support of this, HE [3] stated “*Online teaching may not fully replace on-campus learning”.*
  - According to ELE [1], CSE [2], CHE [1], the online mode of delivery doesn’t allow students and instructors to make the same bond as the f-2-f delivery.
  - INE [2] was more concerned about the assessment process and its fairness when delivered online, stating “*Exams should be held on campus in the future to ensure fairness and eliminate cheating*”.

Table 4 shows a summary of the findings from the instructors’ interviews in relation to the challenge faced and solutions proposed, suggestions for teaching strategies and tools to be used and their reflections upon their online migration experience.

**Table 4:** Summary of the main findings from the instructors’ interviews

*Suggestions*	
Teaching strategies	Focus on more student engagement and class interaction Take the time to learn the provided tools and search for new ones to enhance teaching Take feedback from students Take extra time to prepare and organize online lectures Share your camera as it helps students focus better Empathize with the students and try to understand their point of view Share experiences with everyone so all instructors can benefit Repeat and recap concepts after every chapter; focus on quality over quantity Record lectures
Technology supported learning tools	Blackboard Whiteboard Tool Blackboard Collaborate for 1-on-1 Help Sessions Use the right tools and hardware (ex: headphones, mic, tablet, etc.) Go through assessments using student preview feature in iLearn to prevent unexpected issues Make all exams iLearn based Poll everywhere www.pollev.com used for in class discussion and activities Sword software can be used to write on screen
*Reflections*	
Positive reflections	More flexibility and accessibility especially for graduate level An advantage, not a backup solution IT has done a wonderful job in providing the tools and training for faculty Less noise and distraction in online classes Can be the seed for the blended learning approach Very effective teaching methodology Can be used when Professors are abroad for conferences More suitable for smaller classes
Negative reflections	Undergraduate students would miss out on ‘on-campus experience’ and that the extracurricular activities Seeing the students f–2–f and interacting with them is more academically rewarding and bonding with the students is more tangible It is important to have the assessment process on campus to ensure fairness and eliminate cheating

**Fig. 10:**
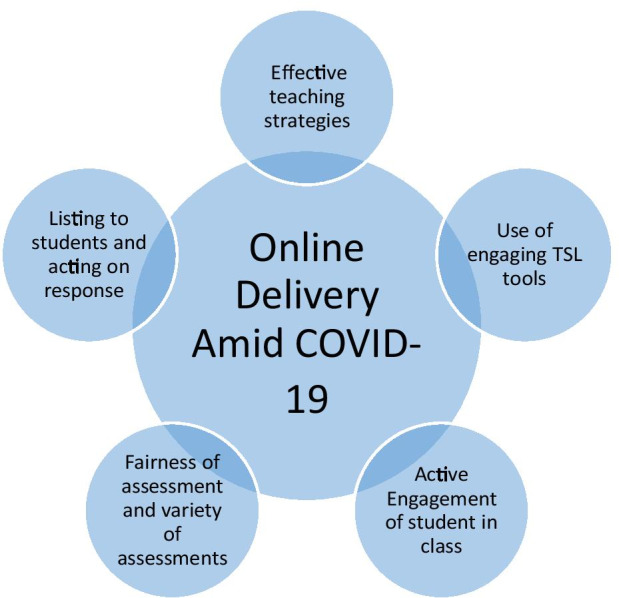
Key elements to successful online delivery amid COVID-19

The findings from this section show that the online delivery of teaching can be made effectives by adopting relevant teaching strategies and the use of more engaging technology supported learning tools, however with the best efforts in place the online delivery has its own benefits and drawbacks that need to be looked into. Figure 10 shows the key elements that were concluded from this study which helped the successful online delivery amid COVID-19.

## Limitations and recommendations for future research

This study focused on a single case study with a small population of engineering students at a selective Higher Education Institution in the UAE, however, the study could benefit from a wider population to examine the challenges faced by other non-engineering discipline in comparison to the engineering discipline. This research was based on exploratory study that targeted engineering students and instructors using surveys and interviews which used mostly descriptive statistical analysis in order to evaluate the status of the sudden migration of online teaching during the COVID-19 pandemic. However, in order to obtain more meaningful insight and reliable results that highlights the status of online delivery over a long prolong period of time, a quantitative study to survey a larger sample of students applying more inferential statistical techniques in order to examine the student’s perceptions of some of the perceived challenges in relation to the background (level of study, program of study, age, gender, discipline etc.) would add more value to the understanding of how to respond to such challenges in future.

## Discussions and conclusions

This study was provoked by the sudden online migration of teaching in academic institutions globally amid convid-19, leaving many of these institutions unprepared for this migration despite the availability of the technology and relevant platforms for synchronous and asynchronous modes of delivery. However, to ensure that the teaching and learning process is conducted effectively, this paper argues that a great deal can be learnt from learning theories in order to use the technology as a tool for effective pedagogies and learning strategies. This paper therefore displayed a consolidated review of learning theories; behavioural, cognitive and experiential learning theories that play different roles in understanding how learning takes place, and how the technology can play a role in developing the different stages of learning. Guided by such understanding, this study evaluated the teaching and learning experiences of students at the selected Higher Education Institution case study as a result of the online migration amid COVID-19. The study concluded that effective learning takes place not only when the online technologies are used to enhance the different stages of the learning process, but also it very much depends on the educators’ attitude and behaviour in accommodating the students’ needs who are under psychological pressure. Hence, when synchronous and asynchronous modes of delivery are used to allow the students engage effectively through well-structured live sessions using tools that mimic f-2-f teaching allowing students engagement and interactions to reinforce the leant concepts, well as having recorded lectures that allow further points of reflection, then the 1^st^ stage of the learning process could be achieved by formulating internalized theoretical concepts that can be tested through suitable methods of assessment. However, it is important to take on board the findings of this study that running online assessments must be conducted in a way that is built on trust while taking all precautionary measures to ensure fairness. Adopting a strategic approach to different methods of assessments that cater for the nature of different engineering subjects, some of which are highly mathematical is crucial. It is also important to note that educators must practice the online delivery tools to perfection in order to maintain the class interest and have contingency plans and backups to deal with other technology failures such as slow internet connections and less responsive platforms.

The study shows that students faced technological, psychological, and pedagogical challenges during the sudden migration online amid Covid-19. It was clear that these challenges impacted on the students learning process due to the lack of preparedness and the ability of the faculty to adapt well to the COVID emergency. Challenges faced by instructors were clearly visible to the students because they were not fully prepared for the online delivery and showed nervousness during teaching and learning. Even though the instructors were not prepared for the sudden migration to the online delivery, they made good effort to mitigated and responded to the COVID-19 impediments. Students showed appreciation of the efforts by the instructors but were not happy with issues like the online examinations/assessments. The full and the expected learning experience of the students were affected by some of these issues. It is therefore important that universities put in place relevant strategies and support systems to face the impact of future pandemics. Finally, the findings of this study showed the teaching pedagogy and the psychological factors posed crucial challenges to the instructors while the impact of these seemed tangible on the students too.

## Data Availability

All the data used in the paper was collected by the authors in compliance with ethical standards.
